# Combined effects of exercise and immuno-chemotherapy treatments on tumor growth in MC38 colorectal cancer-bearing mice

**DOI:** 10.3389/fimmu.2024.1368550

**Published:** 2024-02-15

**Authors:** Manon Gouez, Amélie Rébillard, Amandine Thomas, Sabine Beaumel, Eva-Laure Matera, Etienne Gouraud, Luz Orfila, Brice Martin, Olivia Pérol, Cédric Chaveroux, Erica N. Chirico, Charles Dumontet, Béatrice Fervers, Vincent Pialoux

**Affiliations:** ^1^ Prevention Cancer Environment Department, Léon Bérard Cancer Center, Lyon, France; ^2^ Team Atherosclerosis, Thrombosis and Physical Activity, LIBM EA7424, Université Claude Bernard Lyon 1, Université de Lyon, Faculty of Medicine, Lyon, France; ^3^ Inserm, U1296 Unit, “Radiation: Defense, Health and Environment”, Centre Léon Bérard, Lyon, France; ^4^ Movement, Sport, and Health Sciences Laboratory, EA 1274, Université Rennes 2, ENS Rennes, Bruz, France; ^5^ Institut Universitaire de France, Paris, France; ^6^ CRCL INSERM 1052/CNRS 5286, University of Lyon, Hospices Civils de Lyon, Lyon, France; ^7^ Department of Biomedical Sciences, Cooper Medical School of Rowan University, Camden, NJ, United States

**Keywords:** colorectal cancer, acute exercise, immune check point inhibitor, immune cell, cancer immunotherapy

## Abstract

Acute exercise induces transient modifications in the tumor microenvironment and has been linked to reduced tumor growth along with increased infiltration of immune cells within the tumor in mouse models. In this study, we aimed to evaluate the impact of acute exercise before treatment administration on tumor growth in a mice model of MC38 colorectal cancer receiving an immune checkpoint inhibitor (ICI) and chemotherapy. Six-week-old mice injected with colorectal cancer cells (MC38) were randomized in 4 groups: control (CTRL), immuno-chemotherapy (TRT), exercise (EXE) and combined intervention (TRT/EXE). Both TRT and TRT-EXE received ICI: anti-PD1-1 (1 injection/week) and capecitabine + oxaliplatin (5 times a week) for 1 week (experimentation 1), 3 weeks (experimentation 2). TRT-EXE and EXE groups were submitted to 50 minutes of treadmill exercise before each treatment administration. Over the protocol duration, tumor size has been monitored daily. Tumor growth and microenvironment parameters were measured after the intervention on Day 7 (D7) and Day 16 (D16). From day 4 to day 7, tumor volumes decreased in the EXE/TRT group while remaining stable in the TRT group (p=0.0213). From day 7 until day 16 tumor volume decreased with no significant difference between TRT and TRT/EXE. At D7 the TRT/EXE group exhibited a higher total infiltrate T cell (p=0.0118) and CD8+ cytotoxic T cell (p=0.0031). At D16, tumor marker of apoptosis, vascular integrity and inflammation were not significantly different between TRT and TRT/EXE. Our main result was that acute exercise before immuno-chemotherapy administration significantly decreased early-phase tumor growth (D0 to D4). Additionally, exercise led to immune cell infiltration changes during the first week after exercise, while no significant molecular alterations in the tumor were observed 3 weeks after exercise.

## 1 Introduction

The introduction of new therapies that combine immunotherapy, such as Immune Checkpoint Inhibitors (ICIs) with chemotherapy, has led to a significant improvement in patient survival compared to conventional treatments (1). Despite its acceptable clinical therapeutic efficacy, the primary limitation of immunotherapy lies in innate or acquired resistance to ICIs, which promotes cancer progression and the risk of relapse (2, 3). While chemotherapy acts directly on the tumor, the goal of immunotherapy is to boost the immune system and to restore an effective response against tumor cells. Indeed, promoting a comprehensive physiological environment that enhances immune response could augment the efficacy of immunotherapy.

Physical activity (PA) is now recognized for its benefits on immunity and the reduction of complications related to chemotherapy (4–6). Moreover, post-diagnosis PA is associated with a reduced risk of mortality in cancer patients (7). While the precise mechanisms underlying this beneficial effect are not yet fully understood, several hypotheses have been proposed. It is suggested that PA may enhance tumor vascularization (8–10), and alleviate inhibitory metabolic conditions within the tumor microenvironment, including hypoxia, acidosis, lactate accumulation, and decreased glucose levels (11). Additionally, PA may promote an increased immune infiltration (12–14), contributing to the observed reduction in mortality. Recently, physical exercise has been proposed to enhance the effectiveness of immunotherapy by modulating immune checkpoint inhibitors like PD-1 (Programmed cell death 1) and PD-L1 (Programmed cell death-Ligand 1) (15, 16). Furthermore, studies have observed that exercise conducted immediately before or during chemotherapy infusion in animal cancer models can trigger several advantageous mechanisms, including transient improvements in solid tumor perfusion, reductions in tumor hypoxia, and enhanced drug delivery to tumors (10, 17).

Most of the available evidence on the benefits of physical exercise in cancer patients have been observed in interventions performed either after the treatment or during the interval between the chemotherapy cycles. While published murine model studies have demonstrated a reduction in tumor growth through aerobic exercise training (18, 19), only a limited few have investigated the combination of exercise with immune-chemotherapeutic treatments and no study has evaluated the effect of exercise immediately before treatment administration. Yet, molecular mechanisms underlying the potential direct acute effects of exercise on the immune-chemotherapy effectiveness on tumor remain poorly understood and are still to be studied.

Considering the effects of exercise on both the tumor itself and the systemic immune system, we aimed to evaluate the impact of a pre-treatment administration of acute exercise on tumor development in a mouse model (C57BL/6) of MC38 colorectal cancer treated by ICI and chemotherapy.

## 2 Materials and methods

### 2.1 Cell culture

MC38 murine colon cancer cell line was obtained from Kerafast (USA) and was negative for mycoplasma assays. MC38 cells were cultured in DMEM medium complemented with 10% fetal bovine serum, 100 U/mL penicillin and streptomycin. Cells were incubated in a humidified incubator with 5% CO2 at 37°C.

### 2.2 Animals

All experiments and protocols were compliant with the ARRIVE guidelines and were approved by the Animal Ethics Committee of the University Claude Bernard of Lyon (CEEA: DR2021-44v2) and the Ethics Committee of Centre Léon Bérard Comprehensive Cancer Center (Lyon) (2021-SCAR-107). Six-week-old female C57BL/6 mice (n = 120; Janvier Labs, Le Genest-St-Isle, France) placed in a box equipped with 12:12 inverted light cycle at 22 ± 2°C, were distributed in enriched cages with nest and paper (5-6 mice/cage), with *ad libitum* access to food and water. After reception, mice followed an acclimatization period of one week in the animal house.

### 2.3 MC38 tumor models and tumor monitoring

We adopted the methodology and treatment described in the study by Grasselly et al. (2018). MC38 cells were injected in 6-week-old C57BL/6 mice. Suspensions of exponentially growing cancer cells diluted in 0.2 mL of PBS were injected subcutaneously into the right flank of mice (5.10^6^ cells for MC38). When tumor volume reached 200 mm^3^ (around 5 days), mice were randomized in one of the four groups (1) Control (CRTL), (2) Treatment (TRT), (3) Exercise (EXE) and (4) Treatment plus Exercise (TRT/EXE) and the first treatment was administered (D0). Tumor growths were measured by manual measurement length x width (in mm^3^) using a calliper, every 2 days. To account for a possible loss of body mass, the mice were also weighed 3 times a week. The tumor volume was determined using the formula: V = 4/3 × π × r^3^. In the case of excessive tumor volume (>1600 mm^3^) or if weight loss was too great (15 to 20% in a few days compared with the weight at the start of treatment), the animals were excluded and sacrificed.

### 2.4 Treatment

The combination regimen used for the MC38 colon adenocarcinoma mouse model was composed of capecitabine (Accord), administered *per os* 5 days/week at a dose of 250mg/kg. Oxaliplatin (Accord 5mg/mL) was injected i.p. once a week at a dose of 5 mg/kg. Anti-PD-1 (RMP1-14, BioXCell) was injected i.p. once a week at a dose of 12.5 mg/kg (20).

### 2.5 Exercise protocol

#### 2.5.1 Exercise familiarization and maximal speed assessment

After a one-week acclimatation, mice were familiarized with running on a treadmill (Ugo Basile, Gemonio, Italy) using the following protocol: *day 1*: 5 minutes at 8m/min; *day 2 and 3*: 5 minutes at 8m/min and 10 minutes at 10m/min with no slope. Then, mice performed an incremental speed test to determine their maximum aerobic speed (MAS). The test began with a warm-up period at a speed of 5, 9, 12, and 15 m/min with 15° slope for 5 minutes for each speed. After this period, speed was increased by 2 m/min each 2 minutes. MAS was reached when the mouse stopped running and remained immobile for 5 consecutive seconds on an electrical grid.

#### 2.5.2 Exercise protocol

Mice of the EXE and TRT/EXE groups followed a running program on the treadmill five days per week at 60% MAS for 50 min with no treadmill inclination. The submaximal exercise was scheduled to terminate 15-20 min prior to infusion in TRT/EXE group. CTRL and TRT groups were placed in the room where treadmill running sessions took place, to be exposed to the same stress as EXE mice.

### 2.6 Experiment

In the first set of experiments, mice were trained for 5 days and were sacrificed at 14 days after cancer cell injection. Tumors were dissected and analyzed by flow cytometry (n=20/groups).

In the second set of experimentation, non-treated EXE group (n=8) were trained for 1 week whereas TRT/EXE groups were trained for 16 days. CTRL group (n=8) and EXE group were sacrificed after 12 days of cancer cell injection. TRT (n=12) and TRT/EXE (n=12) groups were sacrificed at 25 days after cancer cell injection. Tumors were dissected and cut in two equal parts at the center; half were frozen in liquid nitrogen and the other half were embedded in OCT prior to being frozen. All samples were stored at −80 °C.

### 2.7 Immune cell phenotyping analyses by flow cytometry

Flow cytometry was used to analyze the tumor immune microenvironment. All tumor tissue samples per group were collected. Cells from the tumors were counted and cell surface markers were stained with the following fluorescently conjugated antibodies: anti-CD45 (30F11, BD Biosciences), anti-CD4 (RM4-5, BD Biosciences), anti-CD8 (53-6.7, Miltenyi Biotec), anti-CD3 (17A2, BD Biosciences), antiCD11b (M1/70, Invitrogen), anti-PD-1 (10F.9G2, BioLegend), anti-CD25 (PC61, Biolegend), anti-CD49b (DX5, BD Biosciences), anti-Granzyme b/Cryofix (GB11, Invitrogen), Viability UV Zombie. Flow cytometry data were acquired on the BD LSRFortessa X20 cytometer and FlowJo software (Ashland, OR, USA) was used for analyses.

### 2.8 Western blot analyses

Tumor samples were lysed in buffer pH 7.4 (10 mM Tris Base, 0.5M sucrose, 50 mM NaCl, 5 mM EDTA, 30 mM Sodium pyrophosphate, 1% NP40, 0.25% sodium deoxycholate, 5µl/ml of inhibitors of proteases cocktail, 50mM NaF and 100µM sodium orthovanadate) and centrifuged at 12 000 g for 12 min (4°C). Protein concentration was determined using Lowry protein assay (Lowry et al., 1951). Proteins (50 μg) were separated by SDS-PolyAcrylamide Gel Electrophoresis (PAGE) and transferred onto nitrocellulose membranes (Bio-Rad, Hercules, CA, USA). The membranes were blocked with 5% BSA or non-fat dried milk in TBS-Tween (0.05%) and were incubated overnight at 4°C with the appropriate primary antibodies (**Table 1**). Then, the membranes were washed three times with TBS-Tween (0.05%) and were incubated with secondary antibodies for 1 hour at room temperature. Immunoreactive bands were visualized with Odyssey Infrared Imaging System (LI-COR Biosciences, Lincoln, NE, USA) and protein loading was normalized to HSC70 expression.

**Table 1:** List of western blot antibodies.

Protein	Molecular weight	Reference
p-ERK1/2	42, 44 kDa	Cell signaling 4376
ERK1/2	42, 44 kDa	Santa Cruz sc-514302
p-AKT	60 kDa	Cell signaling 9271
AKT	60 kDa	Cell signaling 9272
Cleaved caspase 3	17 kDa	Cell signaling 9661
BAX	20 kDa	Cell signaling 2772
BCL-2	28 kDa	Abcam ab7973
HSC70	70 kDa	Santa Cruz sc-7298

### 2.9 RNA extraction and RT-qPCR

Total RNA was extracted from frozen tumor tissue samples using TRIzol^®^ reagent according to the manufacturer’s protocol (Invitrogen, Carlsbad, CA, USA). RNA amounts and purity were determined on a microplate reader (Varioskan Lux, ThermoScientific, Waltham, MA, USA) with a µDrop^®^ plate (ThermoScientific, Waltham, MA, USA) and RNA integrity was controlled on 1.2% agarose gel using the FlashGel electrophoresis system (Lonza, Rockland, ME, USA). Reverse transcription reaction was carried out on 1 μg of total RNA using the iScript Supermix (Bio-Rad, Hercules, CA, USA). QPCR was performed on a CFX-96 Real Time System (Bio-Rad, Hercules, CA, USA) using Sybrgreen method (SsoAdvanced Universal Sybr^®^ Green Supermix, Bio-Rad, Hercules, CA, USA). The expression of target genes was normalized to reference genes (HPRT1, RPL4 and RPL19) and the relative expression was calculated using the ΔΔCt method (**Table 2**).

**Table 2:** List of primers used for RT-qPCR analysis.

Gene	Forward (5′ → 3′)	Reverse (5′ → 3′)
**HPRT1**	AGGCCAGACTTTGTTGGATTT	CAGGACTCCTCGTATTTGCAG
**RPL4**	CGCAACATCCCTGGTATTACT	TGTGCATGGGCAGGTTATAGT
**RPL19**	GAAGGTCAAAGGGAATGTGTTCA	CCTTGTCTGCCTTCAGCTTGT
**Angpt1**	CGTGGAGCCGGATTTCTCTT	TTAGTACCTGGGTCTCAACATCTG
**Angpt2**	TCATCACCCAACTCCAAGAGC	CGGTGTTGGATGACTGTCCA
**IL2rb**	GTCCATGCCAAGTCGAACCT	AGGCGAAGGTTGTCAAAGGG
**IL-6**	ACTTCCATCCAGTTGCCTTCT	GAATTGCCATTGCACAACTCT
**TNFa**	GCCTCTTCTCATTCCTGCTTG	CTGATGAGAGGGAGGCCATT
**CHOP**	CCTGAGGAGAGAGTGTTCCAG	CTCCTGCAGATCCTCATACCA

### 2.10 Statistical analysis

All statistical analyses were performed in Prism9 (GraphPad software, San Diego, CA, USA). All variables were tested for normality using the Shapiro-Wilk test, which tests for normal distribution of the data. The tumor volume data were analyzed using a two-way (Exercise and Treatment) repeated measures ANOVA for experiment 1 and using repeated measures ANOVA (group effect: TRT vs. TRT/EXE) for experiment 2, followed by a Fisher’s Least Significant Difference (LSD) test. Flow Cytometry data (experiment 1) were analyzed using a two-way (Exercise and Treatment) ANOVA, followed by a Fisher’s LSD test. Western Blot and RT-qPCR (experiment 2), independent t-test or Mann-Whitney test was utilized to compare TRT vs. TRT/EXE because distribution was not normal. The comparisons were considered statistically significant for *p* < 0.05. Results were expressed as mean ± SEM (Standard Error of the Mean).

## 3 Results

### 3.1 Exercise prior to administration of immuno-chemotherapy shows an early decrease in tumor growth in the MC38 model of colorectal cancer

**Figure 1:**
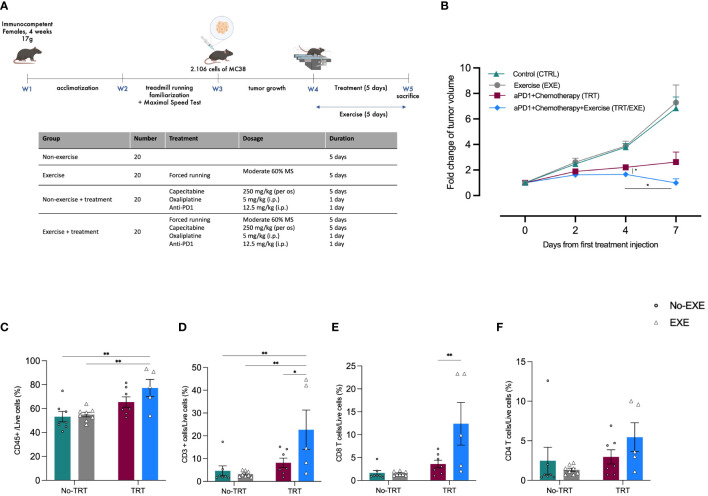
Experimental design and tumor volume change and post intervention intratumoral immune cell infiltration in Experiment 1 **(A)** Experimental study design. N=20 mice/group **(B)** The average fold changes in tumor volume at each measurement were calculated as follows: the ratio of the tumor volume on the corresponding day to the tumor volume on day 0 (Mean ± SEM) **(C-F)** Flow cytometric characterization of MC38 tumor infiltrating immune cell populations: proportion of total in immune infiltration CD45+ cells **(C)**, proportion of total lymphocyte infiltration CD3+ cells on total CD45+ cells (CD45+CD3^+^) **(D)**, proportion of CD4+ T cells on CD3+ (CD45+CD3+CD4^+^) **(E)**, proportion of CD8+ T cells on CD3+ (CD45+CD3+CD8^+^) **(F)** **p<0.01, *p<0.05, TRT: Treatment; EXE: Exercise.

To investigate the impact of acute exercise prior to the administration of a combination of immune checkpoint inhibitors (anti-PD1) and platinum-based chemotherapy, we initially monitored tumor growth over a 7-day period (**Figure 1A**). At Day 0 (D0), the measured tumor volumes were similar among all groups (**Figure 1B**). By Day 7 (D7), exercise alone did not exhibit any effect on tumor growth when compared to the control group (EXE vs. CTRL). In contrast, both the control and exercise groups (CTRL and EXE) showed significantly larger tumors at D7 (**Figure 1B**). However, it is worth noting that a trend toward a more substantial reduction in tumor volume was observed in the EXE/TRT group compared to the TRT group. Specifically, on the final day of Experiment 1 (D7), the mean tumor volume in the TRT group measured 463.5 ± 134.5 mm3, while the EXE/TRT group had tumors measuring 185.5 ± 62.2 mm3 (p=0.0747, **Figure 1B**). Intriguingly, between D4 and D7, tumor volumes remained stable in the TRT group but decreased in the EXE/TRT group, with a significant reduction in tumor volume observed between these two time points (p=0.0213, **Figure 1B**).

### 3.2 Exercise prior to administration of immuno-chemotherapy modulates the infiltration of immune cells in the MC38 colorectal cancer model

We characterized the tumor immune infiltrate profiles by flow cytometry for each group at D7. This characterization encompassed total immune infiltrate CD45+ cells and the total count of CD3+ T cells, including effector cells like CD4+ T cells and CD8+ cytotoxic T cells. The following results are presented as the mean percentages of cells within each treatment group, normalized to the total live cells count.

#### 3.2.1 Total leukocyte infiltrate

As shown in **Figure 1C**, the total leucocyte infiltrate, evaluated by CD45 labeling, was significantly higher in tumors in the TRT/EXE group compared to the CTRL group (72.2 vs 53.2%*; p=0.0063)* and the EXE group (54.9%*; p=0.0094)*. There was no difference between the TRT group (65.9%), EXE group and TRT group.

#### 3.2.2 Total lymphocyte T cells

The total T cells infiltrate, evaluated by CD3 labeling in the TRT/EXE group was significantly higher to the two non-treated groups (p<0.01) and the TRT group (p=0.0118) whereas TRT group was not significantly different than the two non-treated groups (**Figure 1D**). Similarly, CD8+ T cell subpopulations in the TRT/EXE group was significantly higher to the three other groups (p<0.01) whereas TRT group was not significantly different than the two non-treated groups (**Figure 1E**). We found no effect of TRT/EXE on CD4+ subpopulations (**Figure 1F**). The TRT group had significantly less CD8+PD-1+ T cells compared to the CTRL and EXE groups (**Supplementary Data**). EXE also tended to have less CD8+PD-1+ T cells compared to the CTRL group (p=0.06). However, exercise did not have an additive effect on the reduction of CD8+PD-1+ T cells in the TRT/EXE group.

### 3.3 A longer period of repeated bouts of acute exercise prior to administration of immuno-chemotherapy has no additional benefit compared with treatment alone

**Figure 2:**
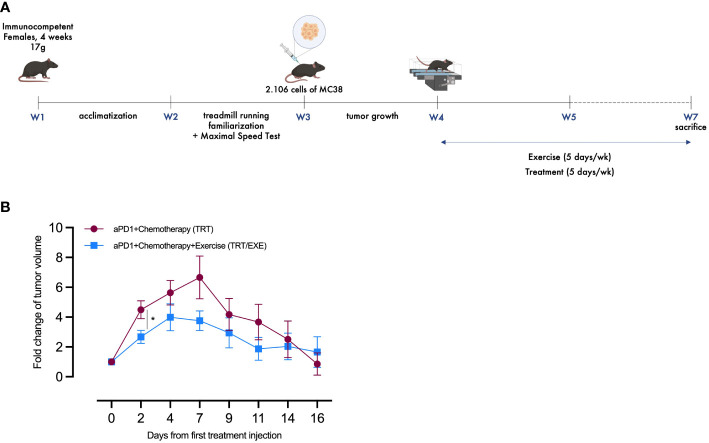
Design and tumor volume change of Experiment 2 **(A)** Experimental study design. N=20 mice/group **(B)** The average fold changes in tumor volume at each measurement were calculated as follows: the ratio of the tumor volume on the corresponding day to the tumor volume on day 0 (Mean ± SEM). All data are presented as mean ± SEM. *p<0.05. TRT: Treatment; EXE: Exercise.

Considering the trends observed in the reduction of tumor growth during Experiment 1, Experiment 2 extended the exercise period in the treated groups to evaluate the kinetics of tumor growth with and without pre-treatment exercise (**Figure 2A**). Similar to Experiment 1, tumor volume was regularly assessed throughout Experiment 2 to evaluate the tumor growth. Data from the CTRL and EXE groups compared to TRT and TRT/EXE confirm Experiment 1, with similar tumor growth between D0 and D7 and the same treatment efficacy (**Supplementary Data**). In the TRT/EXE group, tumor volume tended to increase less compared to the TRT group, with a significant difference observed on D2 (p=0.0245, **Figure 2B**). Additionally, both groups showed a return to volumes close to those at day 0 by day 16, with this effect occurring by day 7 for the TRT group and by day 4 for the TRT/EXE group.

### 3.4 Physical exercise prior to administration of immuno-chemotherapy does not significantly induce changes in the molecular tumor markers

**Figure 3:**
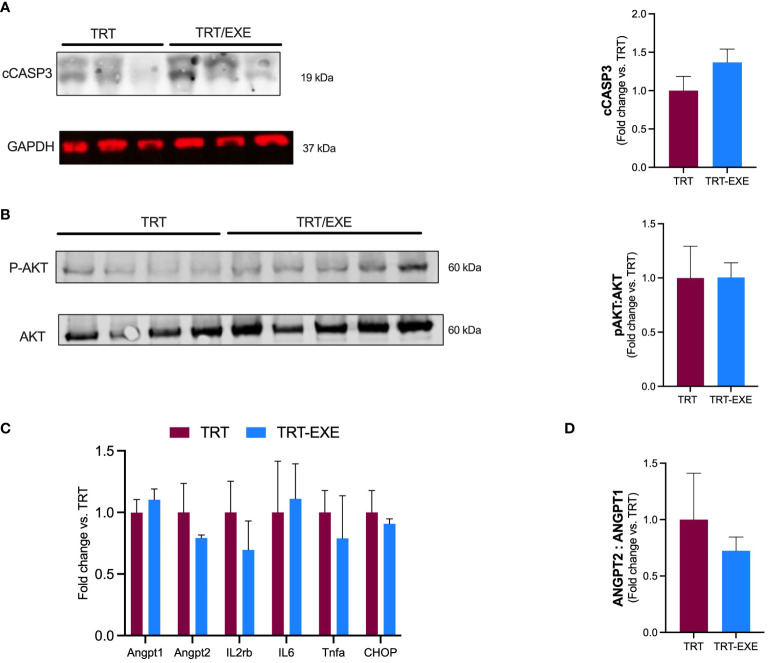
Tumor apoptosis, inflammation, vascular permeability, and growth after 16 days of treatment and exercise **(A, B)** Western blot analysis. Representative and quantification of western blots for cCASP3, GAPDH, pAKT and Akt. **(C)** mRNA expression of angiopoietin-1 (ANGPT1), angiopoietin-2 (ANGPT2), Tumor Necrose Factor alpha (Tnfa), Interleukin 6 (IL6), (CHOP) and Interleukin 2 receptor beta chain (IL2rb) in the tumor. **(D)** ANGPT2: ANGPT 1 ratio. Data are represented as means ± SEM. *p<0.05. TRT: Treatment; EXE: Exercise.

To evaluate the impact of the exercise pre-treatment administration on cell death, we measured apoptosis by immunoblotting of the cleaved caspase-3 (cCASP3). Apoptotic effector cCASP3 protein amount remained unchanged with TRT/EXE compared to TRT (**Figure 3A**). The activation of AKT through its phosphorylation on MC38 tumor cells was evaluated, to assess its promotion of proliferative and survival pathways. The pAKT/AKT ratio showed no change in the group of animals running prior to immune-chemotherapy administration (**Figure 3B**).

Angiopoietin-1 (ANGPT1) and Angiopoietin 2 (ANGPT2) involved in promoting vascular hyperpermeability and weakening of the vascular barrier were not different between TRT and TRT/EXE (**Figure 3C**). We observed that the ANGPT2:1 ratio tended to be lower in the TRT/EXE group than in the TRT group (**Figure 3D**). Il2rβ, a subunit of IL-2 receptor essential for regulation of immune response and expressed by NK cells, was not affected by the combination of exercise-treatment (vs treatment alone). Similar results were observed for Tumor Necrosis Factor alpha (TNF-α) and Interleukine-6 (IL-6) within tumor, 2 cytokine genes coding for pro-inflammatory. Genic expression of CHOP (C/EBP homologous protein-10), a key transcription factor for initiation of apoptotic program under extreme Endoplasmic Reticulum (ER) stress conditions was not different between TRT and TRT/EXE (**Figure 3C**).

## 4 Discussion

Our study is the first to investigate the impact of physical exercise performed just before the administration of immunotherapy with immune checkpoint inhibitor anti PD-1, combined with a platinum-based chemotherapy (oxaliplatin+capecitabine) on the tumor growth in a MC38 colorectal murine model. The primary finding was that, during the early phase of treatment, exercise prior to the administration of immunochemotherapy significantly reduced tumor growth, and significantly increased the total intra-tumoral T-cell infiltrate and CD8+ T cell subpopulations compared to immune-chemotherapy alone. Yet, there was no difference in the final tumor volume (D16) and tumor markers of apoptosis, vascular integrity and inflammation did not significantly differ between these two treatment groups.

### 4.1 A promising antitumor effect with exercise prior to administration of capecitabine-oxaliplatin plus anti-PD1 combination in a MC38 murine model

The significantly slower kinetics of tumor growth in the early stage of MC38 cell development displayed in both experiments by the group receiving exercise in addition to immuno-chemotherapy compared to the group receiving immuno-chemotherapy only, suggests that exercise may enhance the effects of immuno-chemotherapy. However, our second experiment did not support the hypothesis of sustained exercise-induced effects on tumor growth in addition to immunotherapy beyond D7, as the tumor volumes in both groups returned to their initial sizes (D0) on D16. This observation could be the result of the particularly strong effect of this specific immune-chemotherapy combination on MC38 tumor growth, as previously demonstrated, that may overshadow the exercise effect (20). MC38 is a syngeneic colorectal cancer model which has been extensively used in the context of immune checkpoint inhibitor therapy as it has shown robust responses to anti PD1 and anti PDL1 antibodies. Although the subcutaneous (SC) and orthotopic localizations have been shown to differ in terms of tumor immune microenvironment, the SC tumors remain largely used, in particular for the description of resistance mechanisms to immune checkpoint inhibitors (21, 22).

Furthermore, only a few rodent studies combined exercise with immuno-chemotherapy in various clinical cancer settings. Several murine studies have investigated the combination of immunotherapy with exercise in different types of cancer including breast cancer, melanoma liver and lung carcinoma with divergent results (23, 24). Overall, the tumor models (i.e. syngeneic transplanted mouse models, drug-induced or transgenic models) and exercise modalities (i.e. pre-injection *vs* post-injection exercise, programmed *vs* voluntary exercise, intensity) were heterogeneous. In their Patient-Derived Xenograft (PDX) model of non-small cell lung cancer, Martin Ruiz et al. found that combining immunotherapy (specifically nivolumab, a PD-1 inhibitor) with programmed exercise post-injection (aerobic and resistance training, 8 weeks) did not exhibit any additional effect on the inhibition of tumor growth compared to immunotherapy alone (25). In addition, the final tumor volume resulting from the nivolumab + exercise intervention tended to be larger than that observed in the other study groups. The authors attribute this outcome to a ‘paradoxical’ or ‘unconventional’ response associated with the immune cell recruitment and the intratumoral inflammatory environment triggered by these cells. Bay et al. showed an additive effect of a combination of 2 weeks of voluntary wheel running with anti-PD-1 treatment on tumor growth in a B16 melanoma model (26). Gomes-Santos et al. investigated how physical activity, either in conjunction with anti-PD-1 treatment alone or when combined with anti-CTLA-4 treatment, influenced tumor growth in three murine breast cancer models (14). The authors observed that ICI treatment alone had minimal impact on orthotopic breast cancers, whereas combining ICI with programmed treadmill running led to a delay in tumor growth and a reduction in the final tumor volume. Wennerberg et al., found in mice with established 4T1 triple negative mammary carcinoma that combining anti-PD1 and radiotherapy with programmed treadmill exercise significantly slowed tumor growth compared to anti-PD1 and radiotherapy alone (27).

In the present study, the groups without immuno-chemotherapy treatment (CTRL and EXE) exhibited larger tumor size than the treated groups from D4 up to D16, without difference between the two groups. These results confirmed the successful tumor cell implementation and the efficiency of the immuno-chemotherapy treatment previously reported (20). Moreover, the lack of a difference in tumor volume and intratumoral T-cell infiltrate between the control and the exercise only groups suggests that the observed beneficial effect of combined exercise-immuno-chemotherapy might be explained by a potentiation effect of exercise rather than a proper effect of exercise itself.

### 4.2 Exercise before each immuno-chemotherapy administration increases intratumoral T-cell infiltrates

Similar to Grasselly et al. (2020), we reported here that tumor volume reached its peak volume at D7 after treatment initiation and decreased afterwards, in both the TRT and TRT/EXE groups. At D7, we observed a significant increase in total CD45+ leukocyte, CD3+ T lymphocyte and CD8+ T cytotoxic cells infiltration in the combined exercise-immuno-chemotherapy group tumors compared to immune-chemotherapy alone, suggesting that that one week of exercise before immuno-chemotherapy administration is sufficient to increase total leukocyte and T lymphocyte tumor infiltration included the CD8+ cytotoxic T cells. As previously mentioned, exercise is known to have a profound influence on the human immune system (28). Currently, it remains unclear whether the anticancer effects of physical activity are mediated by the acute mobilization and redistribution of cytotoxic effector cells in response to acute exercise sessions or through an adaptive training effect that enhances tissue-associated T cells. In our study, we demonstrated that acute exercise leads to an increase in intratumoral immune cell infiltration, which may explain our observations of reduced tumor growth after 7 days of acute exercise prior to immuno-chemotherapy administration.

To date, several studies in different murine models have observed that exercise without anti-tumor treatment increases intratumoral immune infiltrates after several weeks of treadmill or voluntary wheel running training (12, 27, 29, 30). The results by Wang et al. suggested that a minimum of exercise intensity, comparable to the present protocol, is needed to observe beneficial effects on intratumoral immune cell infiltration (31). Of note, we did not observe an effect of pre-treatment exercise on the infiltration of CD4+ and CD8+ lymphocyte subpopulations. A study showed that training an ApcMin/+ murine model of intestinal cancer on a treadmill for 12 weeks reduced tumor burden and increased CD8+ expression in the tumor (29). Martín-Ruiz et al. (2020) observed that neutrophil tumor infiltration trended to be higher in the exercise group in combination with nivolumab compared to the nivolumab alone group (25).

We hypothesized that exercise combined with anti-PD1 could decrease checkpoint molecule PD-1 expression by CD8+ cells. Our results showed that exercise alone decreases CD8+ cells expressing PD-1+ compared to the control group. However, we found no additive effect on the decrease in CD8+ PD-1+ cell expression when exercise was combined with anti-PD1. Some of the previously mentioned preclinical studies have explored the effects of exercise training on levels of the checkpoint molecule PD-1 expression linked to T cell exhaustion, however results are heterogenous. Wennerberg et al., showed that exercise training, when combined with radiotherapy and anti-PD1 treatment following the injection of 4T1 triple negative breast tumors, reduced the proportion of splenic PD1+CD8+ T cells, which was not achieved by anti-cancer therapy alone, and resulted in reduced tumor growth (27). In their study, Bay et al. showed that voluntary wheel running led to enhanced expression levels of the checkpoint molecule PD-1 and its two ligands PD-L1 and PD-L2 in their B16 melanoma mouse model, without effect on tumor growth with the combination of exercise and anti-PD-1 therapy (26).

Overall, these results suggest that the effects of physical exercise on PD1+ T cells and the efficacy of anti-PD1 immunotherapy are inconclusive. Further research is needed to determine whether the anticancer effects of physical activity are mediated by PD1-dependent or independent pathways.

### 4.3 No molecular changes in MC38 cells with combined exercise and immuno-chemotherapy after 3 weeks of training

Other mechanisms might explain the observed effects of exercise on reducing tumor growth, such as the improvement of tumor vascularization and perfusion, which can lead to a more favorable metabolic profile within the tumor, including the mitigation of hypoxia (8–10, 32–34). Angiopoietin-1 (Angpt1) is an activator of tyrosine kinase receptor TEK expressed mainly on endothelial cells. TEK activation and phosphorylation promote vascular maturity and endothelial cell survival. While Angpt1 counteracts hyperpermeability, Angiopoietin 2 (Angpt2) is upregulated in human cancer and its activation weakens the vascular barrier. Increased ANGPT2/ANGPT1 ratio at the mRNA level has been reported to correlate with neo‐angiogenesis and poor prognosis in many cancer types (35). Unfortunately, we did not find any effect of exercise on the expression of these genes alone, but we have observed a trend towards an increase in the ANGPT2/ANGPT1 ratio. A recent review attributed the difficulty to come to a conclusion on the effect of exercise on vascular remodeling is because of the methodological heterogeneity among preclinical studies (36).

Moreover, McCullough et al. suggested that exercise induces a transient increase in tumor perfusion, potentially leading to enhanced immune infiltration and chemotherapy infiltration within the tumor (10). In the present study, we were not able to explore this hypothesis. Future studies exploring the effect of acute exercise just before or during anti-cancer treatment should plan to measure transient tumor perfusion during exercise.

Furthermore, in the present study, the combination of exercise with immuno-chemotherapy had no effect on apoptosis at D16, whereas we expected an increase in cell death markers such as Caspase 3. One study found a potentiation of apoptosis when exercise was combined with radiotherapy compared with radiotherapy alone in a prostate cancer model (34, 37). In a patient-derived xenograft (PDX) non-small-cell lung cancer model, Martín-Ruiz et al. also found that exercise in combination with PD-1 blockade reduced tumor growth, exhibiting diminished tumor cell proliferation and increase of tumor necrosis. The lack of exercise effect (TRT vs TRT/EXE) on tumor markers of apoptosis, vascular integrity and inflammation is consistent with the absence of a difference in tumor volume at D16.

We did not find any difference in the pAKT/AKT ratio in the tumor, a tumor survival pathway, when exercise was combined with immuno-chemotherapy compared to treatment alone at D16. These results of intratumoral molecular analysis at D16 are consistent with the lack of difference in tumoral volumes observed at that time between the TRT and TRT/EXE groups.

The present study has several limitations. Firstly, the MC38 subcutaneous murine model of colorectal cancer that was used in this study exhibited aggressive tumor growth (22). Most untreated animals reached ethical endpoint criteria, namely a tumor size > 1600 mm3 and tumor necrosis, within 14 days of subcutaneous tumor implantation. Additionally, tumor volumes in the treated groups reached their maximum after 7 days of treatment and exercise. Moreover, it is possible that the impact of the capecitabine+oxaliplatin+anti-PD1 combination was already substantial after a few days, making it difficult to observe additional benefits of exercise when administered alone. For future studies, it would be relevant to investigate the combination of ICI with exercise in less aggressive models of colorectal cancer characterized by slower tumor growth and lower treatment doses. Such models would better mimic the clinical conditions observed in humans.

## 5 Conclusion

We hypothesized that combining PD-1 inhibitors with programmed treadmill running could enhance tumor growth control beyond the effects of immune-chemotherapy alone. Our findings demonstrate that exercise in combination with immune-chemotherapy effectively slows tumor growth in early-stage of MC38 development for up to 7 days after treatment initiation, accompanied by increased tumor immune cell infiltration and particularly CD8+ cytotoxic T cells. However, it is important to note that this study did not provide sufficient evidence to support an additional benefit of exercise when combined with immunotherapy-chemotherapy after a 3-week pre-treatment administration exercise intervention. This may be attributed to the significant anti-tumor impact of the combined therapy used in this study. Consequently, further pre-clinical investigations are warranted to explore the effects of acute physical exercise combined with anti-tumor treatments on tumor growth.

## Data availability statement

The raw data supporting the conclusions of this article will be made available by the authors, without undue reservation.

## Ethics statement

The animal study was approved by Animal Ethics Committee of the University Claude Bernard of Lyon (CEEA: DR2021-44v2) and the Ethics Committee of Centre Léon Bérard Comprehensive Cancer Center (Lyon) (2021-SCAR-107). The study was conducted in accordance with the local legislation and institutional requirements.

## Author contributions

MG: Conceptualization, Data curation, Formal analysis, Funding acquisition, Investigation, Methodology, Project administration, Resources, Software, Validation, Writing – original draft, Writing – review & editing. AR: Investigation, Methodology, Validation, Writing – original draft, Writing – review & editing. AT: Conceptualization, Formal analysis, Methodology, Supervision, Writing – original draft, Writing – review & editing. SB: Data curation, Investigation, Resources, Software, Writing – review & editing. E-LM: Methodology, Writing – review & editing. EG: Conceptualization, Funding acquisition, Investigation, Methodology, Writing – review & editing. LO: Investigation, Methodology, Resources, Writing – review & editing. BM: Data curation, Investigation, Resources, Writing – review & editing. OP: Funding acquisition, Supervision, Writing – review & editing. CC: Conceptualization, Funding acquisition, Investigation, Methodology, Writing – review & editing. EC: Resources, Validation, Writing – review & editing. CD: Conceptualization, Funding acquisition, Methodology, Validation, Writing – original draft, Writing – review & editing. BF: Conceptualization, Funding acquisition, Supervision, Validation, Writing – original draft, Writing – review & editing. VP: Methodology, Writing – review & editing, Conceptualization, Funding acquisition, Supervision, Validation, Writing – original draft.
