# Supplementary material for: Combined effects of exercise and immuno-chemotherapy treatments on tumor growth in MC38 colorectal cancer-bearing mice
Source: Front Immunol. 2024 Feb 15;15:1368550. doi: 10.3389/fimmu.2024.1368550 (PMC10902641; doi:10.3389/fimmu.2024.1368550)
Supplement: Supplementary file 1 [file DataSheet_1.docx]

Supplementary Material

Combined effects of exercise and immuno chemotherapy treatments on tumor growth in MC38 colorectal cancer-bearing mice

Manon Gouez*, Amélie Rébillard, Amandine Thomas, Sabine Beaumel, Etienne Gouraud, Luz Orfila, Brice Martin, Olivia Pérol, Cédric Chaveroux, Erica N Chirico, Charles Dumontet, Béatrice Fervers, Vincent Pialoux

*** Correspondence:** Manon Gouez: [manon.gouez@univ-lyon1.fr](mailto:manon.gouez@univ-lyon1.fr)

# Supplementary Figures

**Supplementary Figure 1.** Flow analyses of PD-1 expression on CD8+ T cells in tumors. Data are shown as Mean + SEM. *p < 0.05 and **p< 0.01 ; ns: non-significant. TRT: Treatment; EXE: Exercise

**Supplementary Figure 2.** Tumor volume in the experiment 2 from D0 to D7. The average fold changes in tumor volume at each measurement were calculated as follows: the ratio of the tumor volume on the corresponding day to the tumor volume on day 0 (Mean ± SEM). All data are presented as mean $\pm$ SEM. *p<0.05. TRT: Treatment; EXE: Exercise
